# Individual perceptions of renewable energy investment in Somali firms

**DOI:** 10.1038/s41598-025-11581-y

**Published:** 2025-08-26

**Authors:** Bile Abdisalan Nor

**Affiliations:** Faculty of Management Science, SIMAD University, Mogadishu, Somalia

**Keywords:** Renewable energy, Theory of planned behaviour, Investment intentions, Somalia, Individual investors, Environmental sciences, Energy science and technology

## Abstract

Somalia’s energy sector is seen as potential for development and investment. financing this sector is crucial for development and economic growth. Small and medium-sized private-sector enterprises are the primary electricity generators and distributors, operating diesel-powered systems via off-grid networks This study investigates the factors influencing investment intentions in renewable energy in Somalia. This study utilized a quantitative research approach employing a descriptive research design. The research data for this study were gathered using a structured questionnaire developed based on previous studies to collect primary data from the targeted respondents. A survey was conducted with 220 potential investors both online and face-to-face methods. The data collected were analysed using SmartPLS. This study’s findings show that the intention to invest in renewable energy is directly determined by attitude towards renewable energy investments, subjective norms, Perceived behavioural control and risk aversion. These findings have important implications for promoting renewable energy in a country with abundant solar, wind, and biomass resources but limited infrastructure and awareness. The practical implications derived from this study suggest that, policymakers and organizations should prioritize campaigns highlighting the advantages of renewable energy, such as energy independence, cost savings, and environmental sustainability. This research contributes to the literature by extending the TPB to fragile states and providing a nuanced understanding of the behavioural and contextual drivers of renewable energy investments.

## Introduction

As the adoption of renewable energy expands globally, investments in this sector are emerging as a viable option for inclusion in investors’ portfolios. Renewable energy investments fall under the category of socially responsible investments, which are investment strategies that incorporate environmental, social, and corporate governance (ESG) criteria to achieve financial returns while creating a positive societal impact. These investments focus on stocks, bonds, and funds of companies engaged in renewable energy businesses. These kinds of investments are crucial in financing renewable energy projects for countries transitioning to a low-carbon economy.

According to the^1^achieving a transition to a low-carbon economy would necessitate investments totalling $7.3 trillion between 2010 and 2030. However^2^ , highlighted those unfavourable global economic conditions in 2020 negatively impacted the level of renewable energy investments. The authors also emphasized that financing renewable energy projects in the future will face significant challenges due to rising government debt, as a substantial portion of funding for such projects has traditionally come from governments, corporate bonds, and large institutional investors. Consequently, the growth of the renewable energy sector cannot rely solely on government funding. Alternative funding sources, such as private investments from individual investors, should be explored to support renewable energy projects.

Since 1990, worldwide electrical consumption has steadily grown, resulting in considerable increases in greenhouse gas emissions. International efforts to decrease them and solve climate change are resulting in widespread electrification of various end users, which increases electricity consumption. As a result, achieving the Sustainable Development Goals for cheap and clean energy requires a significant increase in capital expenditures in renewable power production. Project finance (PF) is an appropriate financing option for significant projects. The financial sector has played a critical role in increasing the use of renewable energy sources for electricity production. Financial institutions like banks are crucial in financing solar, wind, biomass, and geothermal electricity^3^. Thus, financing access in this sector is limited due to a lack of financial system development, notably constraints on funding long-term loans. These constraints have a detrimental effect on small and medium-sized sponsors, given that banking is the key source of funding in developing nations^4^.

Prior studies have applied the theory of planned behaviour (TPB) to examine investment decisions in the renewable energy sectors^5^^6^. They found that it significantly influences households’ intentions to invest in renewable energy projects. The TPB argues that subjective norms, perceived behavioural control, and attitude toward the behaviour influence behavioural intention.

Somalia has significant renewable energy potential. The nation has the potential for many renewable energy sources, including solar and wind power. Because of its location near the equator, it has one of the greatest potentials for onshore wind generation in Africa and one of the highest rates of daily total solar radiation in the word^7^. Despite Somalia’s significant solar energy potential, it remains underutilized^8^. Meanwhile, Investments in renewable energy are multiplying in Africa as the costs of renewable technologies decrease^7^. In Somalia, investment in renewable energy is needed, as it urgently requires affordable and sustainable energy to support its development. The significant national energy deficit presents a strategic opportunity for potential investors. As a key enabling sector, renewable energy can provide cost-effective alternatives to traditional sources such as generators and charcoal, offering promising returns for investors and the government. This study is novel in that it examines the investment environment in Somalia’s renewable energy industry, emphasizing its untapped potential and evaluating the factors influencing the intention to participate in renewable energy in Somalia.

Somalia experiences severe energy insecurity, with a significant portion of its population depending on firewood and charcoal to meet their energy needs^9^. According to the International Trade Administration, most Somali families cook using fossil fuels like charcoal and firewood. Charcoal (47.9 per cent) and firewood (41.3 per cent) are the two most often utilized energy sources, with gas and electricity used rarely^10^. Furthermore, the nation is among the least electrified worldwide. approximately 9 million out of its 15 million people lack access to electricity, making Somalia one of the least electrified in the world^11^. Despite having enormous solar energy potential owing to its proximity to the equator, Somalia’s consumption of solar energy has remained limited due to a lack of energy knowledge, high initial prices, and lack of infrastructure^8^. Additionally, the county’s energy sector is seen as a potential for development and investment, and financing this sector is crucial for development and economic growth. Small and medium-sized private-sector enterprises are the primary electricity generators and distributors, operating diesel-powered systems via off-grid networks. Private Somali enterprises produce around 128 MW, with the majority producing and distributing power independently^12^. Benadir Energy Company (BECO), Somalia’s biggest electric company, built a 10 MW solar farm outside the city and linked it to its power generating and transmission systems^8^. Somalia has significant renewable energy potential. Solar power might create more than 2,000 kWh if the nation achieves maximum capacity. Additionally, Various international organizations and NGOs have also launched renewable energy projects, including the installation of solar panels, mini-grids, and micro-hydro systems, to expand electricity access in underserved regions^13^. Despite these investments, the provision of electricity in Somalia faces numerous challenges, such as a lack of comprehensive regulations, weak government enforcement and regulation and limited financial and capital investment for more significant electricity generation, transmission, and distribution^10^. Moreover, financing renewable energy projects remains a key obstacle since Somalia faces problems in attracting investors due to a lack of trustworthy market data and the country’s uncertain sociopolitical situation^8^. Financing is the most critical barrier to Somalia achieving its potential as a hub for renewable energy^10^.

Existing studies on renewable energy investments have primarily focused on stable economies, with little attention given to fragile states like Somalia^14^. Furthermore, although the TPB has been used to investigate renewable energy adoption in various contexts, its relevance to understanding investment intentions in post-conflict situations is limited. This gap raises crucial questions. What variables impact Somalia’s inclination to invest in renewable energy? How can the TPB be adapted to address the distinct sociopolitical and economic issues such situations face? Additionally, existing research, such as those by^15^  and^13^ , have explored renewable energy in Somalia; however, Behavioral factors influencing investment intentions, such as attitudes towards renewable energy, have not been fully explored in Somalia’s context. Thus, this remains a gap in the literature. This study addresses these gaps by extending the TPB to include contextual factors such as attitudes, subjective norms, perceived behavioural control, and additional factors influencing renewable energy investment intentions, particularly relevant in Somalia.

Due to several critical factors, studying factors that influence intention toward renewable energy investments in Somalia is paramount. First, Somalia experiences severe energy insecurity, with a significant portion of its population depending on firewood and charcoal to meet their energy needs^9^. The country remains one of the least electrified in the world, as approximately 9 million out of its 15 million people lack access to electricity^11^. Secondly, Despite having enormous solar energy potential owing to its proximity to the equator, Somalia’s consumption of solar energy has remained limited due to a lack of energy knowledge, high initial prices, and lack of infrastructure^8^. Finaly, many nations, including Somalia, have concerns about their over-reliance on fossil fuels. Continually, the urge to acquire relatively inexpensive energy by predominantly burning coal is greater than the need to keep the environment in excellent condition^16^.

This study contributes to the literature on factors influencing renewable energy investment in Somalia in several ways. This study expands the Theory of Planned Behavior by applying it to the context of renewable energy investment in Somalia, a setting that has remained largely unexplored. This extension provides a more nuanced understanding of renewable energy investment intentions’ behavioural and contextual drivers in post-conflict settings. This research also provides valuable insights to support East African countries in achieving the Sustainable Development Goals (SDGs), commonly called global goals. We also add to the literature by analyzing the consequences of burning fossil fuels, which releases large amounts of carbon dioxide, a greenhouse gas, into the air. We also discuss the advantages of renewable energy investment. Renewable energy investment reduces overreliance on fossil fuels, which is the primary driver of climate change. Furthermore, this study offers practical insights to support the design and implementation of effective policies and strategies to promote corporate engagement in renewable energy practices to attain sustainable goals. This study provides critical theoretical implications by demonstrating that the intention of renewable energy investments is directly determined by Attitude, subjective norm, Perceived behavioural control and risk aversion. The confirmation of the hypotheses (H1, H2, H3 and H4). Our results are consistent with the TPB literature in that they support the role of attitude, subjective norm, PBC, and risk aversion in defining investment intentions.

The organization of the paper begins with an introduction in section one. A literature review follows this in section two. Section three discusses the research methodology. This is followed by section four on findings. Sections five and six discuss the study’s results and conclusions, respectively.

## Literature review

### Theoretical literature review

The theory of planned behaviour (TPB) will serve as the leading theory for this study. Icek Ajzen created the Theory of Planned Behavior (TPB) to forecast human behavior^17^. The TPB argues that subjective norms, perceived behavioural control, and attitude toward the behaviour influence behavioural intention.

The first construct of the theory is the behavioural intention, which refers to the motivational factors that affect behaviour^17^. As the intention to engage in a specific behaviour increases, the likelihood of performing that behaviour increases. Behavioural intention is regarded as a causal and closest mechanism that influences evidence-based practice (EBP) utilisation. Various methods have been employed to measure intention in implementation studies, and it is uncertain which method is the most predictive^18^. The second construct is the attitude toward behaviour, which refers to the degree to which an individual assesses a specific behaviour favourably or unfavourably. Behavioural beliefs and outcome evaluations comprise attitude. The attitude toward behavioural intention has been demonstrated to be significant in a variety of studies, including green purchasing behavior^19^ and investment intention^20^. The third construct is the subjective norm, which is social pressure to perform or not perform a specific behaviour. Subjective norms result from the combination of motivation to comply and normative beliefs. Perceived behavioural control is also a critical component of the TPB, and it pertains to individuals’ assessments of the ease or difficulty of engaging in the behaviour of interest. A subjective norm was demonstrated to influence an individual’s behavioral intention in collectivist countries significantly^21^. Somalia is one of collectivist countries.

TPB is a psychological theory developed to explain individual decision-making and behaviour. However, it can be applied within organisational contexts^22,23^. For instance^22^, Offers research using the theory of planned behaviour (TPB) to investigate environmental behavioural intentions within a workplace context. Their findings indicate that TPB components accounted for 46–61% of the variation in employees’ intentions to participate in three environmental behaviours.

Research on the TPB has made significant progress since the theory was introduced approximately two decades ago. The initial studies primarily evaluated the theory’s predictive validity in various behavioural domains. The theory is supported by the combined weight of a significant amount of empirical evidence, which is best captured in meta-analytic syntheses such as the one included in the current set of articles^24^.

### Empirical review

Attitude involves individuals feeling either favourable or unfavourable towards a particular behaviour. The aspect of Attitude under the theory of planned behaviour is linked to the consumer’s behaviour towards adopting renewable energy. A previous study highlighted that Attitude significantly influenced investors’ intention to invest in renewable energy. For instance^25^, did a study using questionnaire based on the TPB theory found that contextual factors such as attitudes, subjective norms and perceived behavioural control positively affect renewable energy investment intentions in Australia. The results of this study suggested that the respondents’ overall perception of the scheme’s advantages was favourable, and it was the most significant factor in determining their intention to invest, rather than their capacity to invest or duress from others^6^. supported this result and urged that attitudes and other contextual factors positively influence individuals’ intention to invest in renewable energy in Malaysia. Individuals in Malaysia are aware of the significance of environmental preservation, which results in a positive attitude toward environmentally friendly initiatives. Furthermore^26^, revealed that individuals in China possess a positive attitude and demonstrate a significant level of interest in renewable energy. They are concerned about environmental issues and have stated that renewable energy offers numerous advantages over traditional electricity, including preserving the environment from carbon emissions and enhancing energy structures. However, few studies includes ^2828^ showed that attitudes to renewable energy had no effect on intention to use renewable energy. Hence, we propose the following hypothesis:

H1: Attitudes towards renewable energy has a significant impact on individual’s intention to invest in renewable energy.

The relationship between perceived behavioural control and an individual’s intention to invest in renewable energy has been extensively studied within the TPB framework^28^. utilized the theory of planned behaviour (TPB) and the protection motivation theory (PMT) to ascertain the primary factors that influence the environmental behaviour and intentions of farmers in China concerning NPS pollution. Results demonstrated that intention was substantially influenced by the subjective norm, attitude, and self-efficacy (i.e., perceived behavioural control). A study conducted by^29^ demonstrated that subjective norms and perceived behavioural control significantly influenced the intention to use green energy. The results indicate that these variables can positively impact green energy intention, which can be used to develop effective social marketing strategies and comprehensive political initiatives, such as training and awareness campaigns. Hence, we propose the following hypothesis:

H2: Perceived behavioral control has a significant impact on an individual’s intention to invest in renewable energy.

Subjective norms are one of the key drivers of renewable energy investment. Previous research highlighted that a person’s perception of social expectations influences their intention to use renewable energy^27^. showed that subjective norms significantly and positively determined the intention to use renewable energy in Lithuania, a Central and Eastern European country (CEE). This suggests that individual’s perception in Lithuania influences their intention to use renewable energy. Similarly^30^, show that subjective norms have an impact on the attitude towards the use of renewable energy sources^6^. supported this result and urged that subjective norms and other contextual factors positively influence an individual’s intention to invest in renewable energy in Malaysia. Hence, we propose the following hypothesis:

H3: Subjective norms have a significant impact on an individual’s intention to invest in renewable energy.

Risk aversion refers to an individual’s attitude to avoid risk. The literature highlights the relationships between Risk aversion and the intention to invest in renewable energy. This explains why investors are unwilling to invest in renewable energy when there is high risk. To assess the determinants of householders’ investment intention in renewable energy (RE) projects^31^, extended the theory of planned behaviour (TPB) model. The primary data was collected from Iranian households (*n* = 280) using random sampling through structured questionnaire interviews. According to the analyses using structural equation modelling (SEM) and Amos software, attitudes, subjective norms, perceived behavioural control, and perceived risk substantially influence households’ intentions to invest in RE projects. This study also emphasizes that green finance instruments are essential in facilitating private sector investment in renewable energy projects. however^32^, found that there is a negative relationship between Risk aversion and energy-related investments. Similarly^6^, supported this result and urged that Risk aversion negatively influences an individual’s intention to invest in renewable energy in Malaysia. These studies indicate that households with stronger risk aversion are less likely to invest in renewable energy. Hence, we propose the following hypothesis:

H4: Risk aversion has a significant impact on an individual’s intention to invest in renewable energy.

**Fig. 1:**
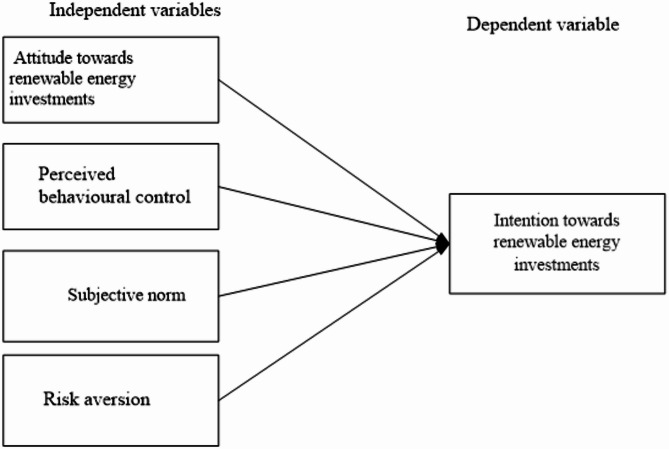
Research framework, Source: Figure by authors.

In Fig. 1, we present the research framework guiding this survey, illustrating the Factors that influence the Intention towards renewable energy investments in Somalia.

## Materials and methods

### Study design

**Fig. 2:**
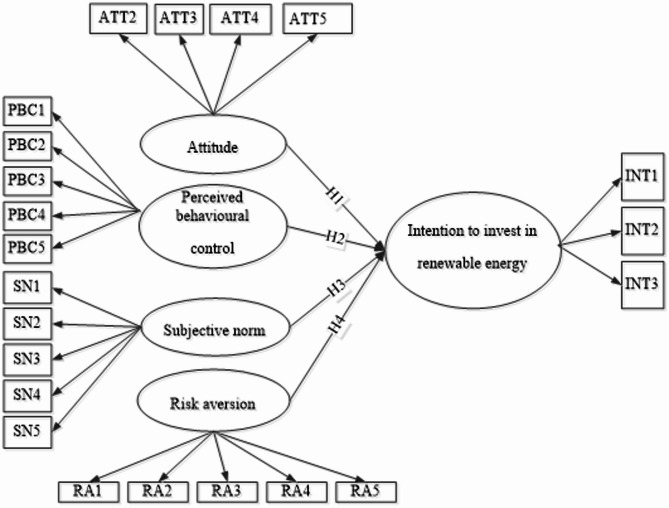
Theoretical conceptual model. Source: Figure by authors.

A comprehensive empirical study is proposed to address the identified research gaps. To ensure accuracy, this research used purposive sampling to target individuals with investment experience, which aligns with the objective of our study. The sample size for this study is 300. The recommended minimum sample size for this study is 92, which was calculated using G*power, a program to calculate the required sample size recommended by^33^. However, the data study centres on 220. According to^34^. A minimum sample size ensures the model’s results are robust and generalisable. Thus, the final sample size for this study is 220. For data analysis, structural equation modelling was implemented; consequently, the theoretical model is illustrated in Fig. 2 using the path model.

### Survey instrument and data collection

A structured questionnaire was employed to gather data from individuals in Somalia regarding their intentions towards renewable energy investments. The questionnaire was designed to align with the extended Theory of Planned Behaviour (TPB) framework, incorporating attitudes, subjective norms, perceived behavioural control, and additional factors influencing renewable energy investment intentions.

Before the main study, a pilot test was conducted using a convenience sample of 50 respondents not included in the primary survey. All the variables are confirmed to be reliable in that the Cronbach’s alpha of the variables such as Attitudes towards renewable energy investments, Subjective norms, Risk aversion and Intention to invest in renewable energy become 0.742, 0.843, 0.814, 0.805 and 0.719, respectively. Data was collected between December 2023 and March 2024 in Mogadishu, Somalia. The research data for this study were gathered using a structured questionnaire developed based on previous studies to collect primary data from the targeted respondents. The questionnaires were distributed to individuals aged 18 and above who were potential investors or had expressed interest in renewable energy. All methods were conducted according to relevant guidelines and regulations. The research protocol, survey tools, and consent process were approved by the Ethics Committee of the Centre for Research and Development at SIMAD University (Approval Number: EC000137). Informed consent was obtained from all respondents before data collection. Participation was voluntary, and all respondents were assured of the confidentiality and anonymity of their responses.

### Measurement of variable

The questionnaire used in this study incorporated scales slightly modified from previous research and is summarised in Table 1 to measure the variables related to promotional mix elements and tourism marketing performance. These variables were grouped into five categories, each drawing on established literature.

**Table 1:** Measurement of the latent constructs.

Constructs	Items	Label	Measurement items	Source
Attitude		ATT1	When investing, I prioritize choosing companies that align with ethical and socially responsible principles.	^ 35^
		ATT2	When investing, I focus on companies that prioritize environmental sustainability.	
	5	ATT3	When investing, I would reject higher returns which are not in favour of sustainability aspects.	
		ATT4	When investing, I carefully assess a company’s environmental compatibility.	
		ATT5	When investing in a company, it should be ecologically harmless.	
Subjective norm		SN1	Most people who are important to me think I should invest in renewable energy.	^36^
	5	SN2	Most individuals whose opinions I respect would encourage me to invest in renewable energy initiatives.	
		SN3	The level of influence from my family can significantly impact my decision regarding investments in renewable energy.	
		SN4	The degree of influence from my friends can greatly impact my decision-making regarding investments in renewable energy.	
		SN5	The level of influence from my colleagues can significantly shape my decisions regarding investments in renewable energy.	
Perceived behavioural control		PBC1	I am confident in my ability to participate in renewable energy investments.	^37^
		PBC2	I am capable of overcoming any obstacles or challenges that might hinder my ability to engage in renewable energy investments.	
	5	PBC3	Participating in renewable energy investments is something I can manage on my own.	
		PBC4	Participating in renewable energy investments is straightforward.	
		PBC5	I have adequate knowledge to engage in renewable energy investments.	
Risk aversion		RA1	The risk of losing money on the investment causes mental stress	^35^
		RA2	The stability of my investments is more important to me than than the possibility of quick profits.	
	5	RA3	The continuity of my investments is more important to me than the opportunity for quick profits.	
		RA4	Even small financial losses make me nervous.	
		RA5	I am hesitant to take risks when it comes to financial decisions.	
Intention to invest in renewable energy		INT1	I expect to invest in renewable energy	^36^
		INT2	I would invest in renewable energy whenever I am given the opportunity.	
		INT3	I will make an effort to invest in renewable energy in the near future.	
	3			

### Data analysis

The data gathered were analyzed utilizing SmartPLS version 3, a sophisticated statistical software designed for partial least squares structural equation modelling (PLS-SEM), which is well-suited for investigating the relationships among variables within the study. The validity and reliability of the measurement scales were evaluated through confirmatory factor analysis (CFA), while hypotheses were examined using structural equation modelling (SEM). Consequently, this research utilized a measurement model (CFA) to assess the validity and reliability of the data. Additionally, SEM was employed to quantify and test the hypothesized relationships among attitudes towards renewable energy investments, subjective norms, risk aversion, and the intention to invest in renewable energy.

## Results and discussions

### Respondents’ profile

This study measures Factors that influence Intention towards renewable energy investments in Somalia: extending the theory of planned behaviour. A total of 220 respondents were obtained for analysis.

**Table 2:** Demographic characteristics of the respondents.

Category	Frequency	Percentage (%)
Gender		
Male	130	50.1
Female	90	49.9
Total	**220**	**100**
**Age**		
Below 21 years	45	20.5
21–30 years	60	27.2
31–40	95	43.2
41–50	20	9.1
Above 50	0	0
Total	**220**	**100**
**Education Background**		
Diploma	50	22.7
Undergraduate Degree	75	34.1
Postgraduate Degree	65	29.6
Others	30	13.6
Total	**220**	**100**
**Occupation**		
Professional	12	5.5
Manager/senior manager	50	22.7
Executive	40	18.2
Non-Executive	18	8.2
Self-employed	70	31.8
Others	30	13.6
Total	**220**	**100**

Table 2 presents the summary of the respondents’ demographic profile statistics. There are 50.1% male and 49.9% female respondents, indicating a nearly balanced gender representation. This balance ensures that both male and female perspectives are equally reflected in the study findings. Within the sample, most respondents are between the ages of 31 and 40 (43.2%), followed by those aged 21 to 30 (27.2%). Respondents below 21 years constitute 20.5%, while those aged between 41 and 50 form 9.1%. Notably, there are no respondents above 50 years. This distribution highlights that younger and middle-aged adults are the primary contributors to the study, likely due to their active participation in investment-related activities. Regarding education, most respondents hold undergraduate degrees (34.1%), followed by postgraduate degrees (29.6%). Respondents with diplomas account for 22.7%, while those with other educational qualifications make up 13.6%. This indicates that most respondents have attained higher education, suggesting an informed and knowledgeable sample that can understand and evaluate renewable energy investments. Regarding occupation, the largest group comprises self-employed individuals (31.8%), followed by managers or senior managers (22.7%) and executives (18.2%). Professionals account for 5.5%, while non-executives make up 8.2%. The remaining 13.6% are categorized as “Others,” likely representing students, homemakers, or retirees. The dominance of self-employed individuals and those in managerial roles suggests that the study captures insights from decision-makers and entrepreneurs who may have a greater inclination toward renewable energy investments.

### Assessment of measurement model

The research used a quantitative approach, employing structural equation modelling. The scale’s validation is based on the quality criteria, reliability, and validity of the constructs to evaluate the internal consistency and convergent validity. The model is evaluated using Cronbach’s alpha, the composite reliability index (CRI), and the average variance extracted (AVE). For Cronbach’s alpha^38^, recommends a minimum value of 0.70, while^39^ recommends values exceeding 0.70 and 0.5 for CRI and AVE, respectively.

As shown in Table 3, all constructs meet the specified criteria. Based on this premise, it is feasible to confirm that the model has convergent validity and scale reliability. In addition, as suggested by Hair^40^the elements must have a factor loading greater than 0.5. This condition was met for all items except ATT1. However, ATT1 was removed from the measurement model because removing the indicator improved other indicators’ values.

**Table 3:** Factor loadings, reliability, and convergent validity assessment.

	Items	Factor loading	Cronbach’s alpha	Composite reliability (rho_a)	Average variance extracted (AVE)
	ATT2	0.699			
Attitude	ATT3	0.757	0.724	0.732	0.545
	ATT3	0.725			
	ATT5	0.769			
Intention to invest in renewable energy	INT1	0.839	0.719	0.731	0.639
	INT2	0.778			
	INT3	0.779			
Perceived behavioural control	PBC1	0.786	0.814	0.815	0.574
	PBC2	0.757			
	PBC3	0.739			
	PBC4	0.720			
	PBC5	0.783			
Risk aversion	RA1	0.732	0.806	0.810	0.563
	RA2	0.785			
	RA3	0.777			
	RA4	0.738			
	RA5	0.717			
Subjective norm	SN1	0.716	0.843	0.854	0.615
	SN2	0.835			
	SN3	0.754			
	SN4	0.817			
	SN5	0.794			

**Table 4:** Discriminant validity using HTMT criterion.

	Attitude	Intention to invest in renewable energy	Perceived behavioural control	Risk aversion	Subjective norm
Attitude					
Intention to invest in renewable energy	0.813				
Perceived behavioural control	0.823	0.839			
Risk aversion	0.847	0.827	0.802		
Subjective norm	0.829	0.836	0.807	0.910	

**Table 5:** Discriminant validity using the Fornell–Larcker criterion.

	Attitude	Intention to invest in renewable energy	Perceived behavioural control	Risk aversion	Subjective norm
Attitude					
Intention to invest in renewable energy	0.813				
Perceived behavioural control	0.863	0.819			
Risk aversion	0.827	0.927	0.742		
Subjective norm	0.849	0.846	0.507	0.680	

Note: The values on the diagonals indicate the square root of the AVE, whereas the off-diagonal values represent the correlations between constructs.

Discriminant validity testing has become a widely acknowledged precondition for investigating relationships between reflectively assessed constructs. The hetero trait-mono trait (htMt) ratio and the Fornell-Larcker criteria were employed to evaluate discriminant validity. Such assessments consider the distinctness of different notions inside the measurement model^41^.

^[[42]]^ proposed measuring the HTMT correlation ratio to a threshold of less than 0.85 to determine discriminant validity. As seen in Table 4, all HTMT values are less than 0.85, showing high discriminant validity. Furthermore, discriminant validity was assessed using the Fornell-Larcker criteria. Discriminant validity is obtained when the square root of each construct’s average extracted variance (aVe) exceeds the correlations between that construct and other constructs^39^. Table 5 shows that the diagonal components indicate each construct’s square roots of the aVe. These results were much higher than the correlations shown below, indicating that discriminant validity was established in the evaluations (See Table 5).

### Structural model analysis

Table 6 summarizes the structural model assessment using essential indicators such as R-square (R²), Adjusted R-square, F-square (f²), Q-square (Q²), and Variance Inflation Factor (VIF). Intention to Invest in Renewable Energy has an R² value of 0.573, indicating that independent components account for 57.3% of the variance. The adjusted R² of 0.565 demonstrates the model’s resilience given the amount of predictors. The F-square (f²) values show the influence of independent constructs on the dependent variable. The findings show that Perceived behavioural control (0.026),), Risk Aversion (0.055) AND Subjective Norm (0.034) have minor effect sizes, reflecting a minimal influence on Intention to Invest. Attitude with an f² of 0.016 has a negligible impact size. These results indicate that the predictors’ individual influence is modest despite their contribution to explaining the intention. The Q-square (Q²) value for Intention to Invest (0.349) is positive, confirming the model’s predictive validity. The Variance Inflation Factor (VIF) values for Attitude (2.134), Perceived Behavioural Control (3.224), Risk Aversion (3.958), and Subjective Norm (3.385) are all less than 5, suggesting significant multicollinearity issues in this model. Overall, the model has strong explanatory power and predictive relevance, with Risk Aversion showing as the most influential predictor.

**Table 6:** Structural Model.

	*R*-square	*R*-square adjusted	F-square	Q-square	VIF
Intention to invest in renewable energy	0.573	0.565		0.349	
Attitude			0.016		2.134
Perceived behavioural control			0.026		3.224
Risk aversion			0.055		3.958
Subjective norm			0.034		3.385

### Hypothesis testing

The findings presented in Table 5 explore the factors influencing individuals’ Intention to Invest in Renewable Energy. The results are based on hypothesis testing, likely using structural equation modelling (SEM) or regression analysis.

**Fig. 3:**
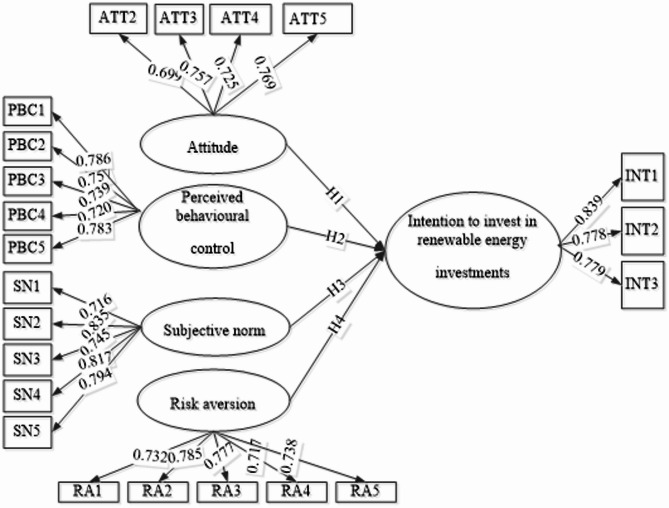
Structural model for this study Source: Figure by authors.

This research formulated four hypotheses to examine the relationships between the variables under consideration. Each hypothesis was thoroughly statistically tested to establish its significance and contribution to the model. The structural model results shown in Fig. 3 and detailed in Table 5 give valuable insights into the connections between the constructs, shedding light on the underlying dynamics driving the phenomena under investigation.

Regarding the findings presented in Table 5, the attitude has a positive and statistically significant effect on the intention to invest in renewable energy (*p* < 0.1). This result indicates that for every unit change in attitude toward renewable energy investment, an individual’s intention to invest in renewable energy increases by 0.119 units (see Table 7). This research aligns with behavioural finance, which states that if an individual’s attitude becomes positive, their investment intentions will likely increase. Thus, this result fully supports H1 and is in line with^6^.

Perceived Behavioural Control (PBC) has a substantial and statistically significant positive effect on investment intentions (*p* < 0.05). Perceived behaviour control assesses an individual’s competence and understanding to conduct a specific behaviour. This research uses investors’ knowledge of impact investing, their moral beliefs, and their financial capacities to invest in renewable energy investment. This result indicates that for every unit change in attitude toward renewable energy investment, an individual’s intention to invest in renewable energy increases by 0.188 units (see Table 7). Thus, this result fully supports H2, and it is in line with^28^.

Subjective Norm also has a significant positive effect on investment intentions (*p* < 0.05). The coefficient of 0.306 (see Table 7) suggests that social pressure or the influence of friends, family, and society significantly impacts people’s intentions to invest in renewable energy. Thus, this result fully supports H3, and it is in line with^43^.

The result provided that risk aversion also has a significant positive effect on investment intentions (*p* < 0.05). The coefficient of 0.222 (see Table 7) suggests investors with a high level of risk are more likely to make more investments. Thus, this result fully supports H4, and it is in line with^44^. This finding seems to contradict the findings arrived at by^32^ Who found that there is a negative relationship between Risk aversion and energy-related investments. This is due to Somalia’s unique circumstance of a fragile state, where traditional investments are often more hazardous due to political instability and underdeveloped infrastructure. In these situations, renewable energy, particularly solar energy, is becoming a safer and more stable option, sometimes with the help of NGOs or foreign donors. Risk-averse investors would thus consider solar energy investments as a safe alternative compared to other investments.

**Table 7:** Findings of hypothesis tests.

Hypothesis	Statement	Original sample (O)	Sample mean (M)	Standard deviation (STDEV)	T statistics (|O/STDEV|)	Result	*P* values
H1	Attitude -> Intention to invest in renewable energy	0.119	0.123	0.067	1.769	Supported	0.077
H2	Perceived behavioural control -> Intention to invest in renewable energy	0.188	0.185	0.095	1.983	Supported	0.047
H3	Subjective norm -> Intention to invest in renewable energy	0.222	0.226	0.097	2.282	Supported	0.023
H4	Risk aversion -> Intention to invest in renewable energy	0.306	0.306	0.089	3.451	Supported	0.001

## Discussions

The world is addicted to fossil fuels. From the lighting in our homes to the fuel in our cars, the energy we use daily is primarily powered by fossil fuels, the main drivers of climate change. Furthermore, many nations, including Somalia, are over-reliant on fossil fuels. Burning fossil fuels releases large amounts of carbon dioxide, a greenhouse gas, into the air. In turn, greenhouse gases blanket the Earth and trap the sun’s heat, warming the planet with far-reaching consequences for people and the planet. Investing in renewable energy reduces all these challenges. Renewable energy investment reduces overreliance on fossil fuels, which is the main driver of climate change.

This research analyses investors’ intentions to invest in renewable energy in Somalia. TPB was employed as a theoretical framework for this study, focusing on intentions for renewable energy investments, which have not been thoroughly studied in prior studies. The findings of this study provide valuable insights into the factors influencing individuals’ intentions to invest in renewable energy. The results demonstrate that Attitude, Perceived Behavioural Control (PBC), Subjective Norm and Risk Aversion are significant predictors of individuals’ Intention to Invest in Renewable Energy in Somalia. These findings have important implications for promoting renewable energy in a country with abundant solar, wind, and biomass resources but limited infrastructure and awareness.

The Attitude towards renewable energy investment has a positive and statistically significant effect on the intention to invest in renewable energy, with an associated P-value = 0.077 and a coefficient = 0.119 (Table 7). This result indicates that for every unit change in Attitude toward renewable energy investment, an individual’s intention to invest in renewable energy increases by 0.119 units, thereby confirming the hypothesis. Attitude’s positive and statistically significant effect emphasizes the significance of promoting favourable attitudes toward renewable energy. This research aligns with behavioural finance, which states that if an individual’s Attitude becomes positive, their investment intentions will likely increase. Research has shown potential for investment in renewable energy based on one’s attitude toward renewable energy investments^6^. In Somalia, where knowledge and awareness of renewable energy technologies are still growing, favourable views may significantly impact investment intentions. This study implies that educational initiatives to change public opinions of renewable energy can considerably increase investment intentions. People in Somalia are more likely to invest in renewable energy. Thus, the Somali government should encourage private investors by establishing regulations and tax credits favourable to such investments.

Perceived Behavioural Control (PBC) has a substantial and statistically significant positive effect on investment intentions with an associated P-value = 0.047 and a coefficient = 0.188 (Table 7). Perceived behaviour control assesses an individual’s competence and understanding to conduct a specific behaviour. This research uses investors’ knowledge of impact investing, their moral beliefs, and their financial capacities to invest in renewable energy investment. This result indicates that for every unit change in Attitude toward renewable energy investment, an individual’s intention to invest in renewable energy increases by 0.300 units, confirming the hypothesis. The TPB model posits that investors’ confidence in their controllability and ability to invest in renewable energy will influence their intention to invest. The significant influence of PBC emphasizes the importance of empowering individuals with the knowledge, skills, and resources they need to invest in renewable energy. Microfinancing, community-based renewable energy projects, and simplified investment mechanisms can improve perceived control and encourage participation. Moreover, the positive impact of perceived behaviour control on investment intention indicates that the desire to invest is increased proportionately to the degree of behavioural control shown and the ease with which it is carried out.

Subjective Norm also has a significant positive effect on investment intentions with an associated P-value = 0.023 and a coefficient = 0.222 (Table 7). The coefficient of 0.222 suggests that social pressure or the influence of friends, family, and society significantly impacts people’s intentions to invest in renewable energy, thereby confirming the hypothesis. A subjective norm is a person’s perspective on a certain conduct that is affected by the perspectives of others. The effect of the Subjective Norm demonstrates the importance of social influence in shaping investing intentions. Research has demonstrated that an individual’s behaviour is shaped by others’ expectations and subsequently influenced by these perceptions^45^. In Somalia’s collectivist society, where collective decision-making is shared, community leaders, religious figures, and prominent organizations’ support of renewable energy might dramatically increase adoption. Using social networks and community-driven projects may help foster trust and involvement. Furthermore, Somali people are self-motivated and trust each other. The degree of influence from a friend or family can significantly impact an individual’s decision-making regarding investments in renewable energy. Somali subjective norms are essential to an individual’s intention to invest in renewable energy.

The result provided that risk aversion also has a significant positive effect on investment intentions, with an associated P-value = 0.001 and a coefficient = 0.306 (Table 7), thereby confirming the hypothesis. This suggests investors with high level of risk are more likely to make more investments. This finding suggests that individuals who demonstrate overconfidence are more likely to make investment decisions that are influenced by their exaggerated self-belief in their own capacity. In Somalia, risk aversion is an important factor that positively influences investment intention. This is due to the high level of uncertainty in the country, which can result in a preference for low-risk investments and saving. Examples of such uncertainties include climate change, conflict, and instability. Moreover, due to Somalia’s unique circumstance of a fragile state, where traditional investments are often more hazardous due to political instability and underdeveloped infrastructure, renewable energy, particularly solar energy, is becoming a safer and more stable option, sometimes with the help of NGOs or foreign donors. Risk-averse investors would thus consider solar energy investments as a safe alternative compared to other investments.

### Theoretical implications

The main contribution of this study lies in its capacity to offer a holistic and detailed vision that enriches the understanding and implementation of sustainability strategies in renewable energy investment. This work expands the Theory of Planned Behaviour to the context of fragile states by applying it to the context of renewable energy investment in Somalia, a setting that has remained largely unexplored. This extension provides an understanding of the behavioural and contextual drivers of renewable energy investment intentions in post-conflict settings. The study specifically examines how the model’s core constructs, Attitude, Subjective Norms, and Perceived Behavioural Control, operate within environments characterised by weak institutions, political instability, and limited infrastructure. In fragile states such as Somalia, not only social pressure and individual attitudes but also structural constraints, including limited access to credible information, insecurity, and a lack of regulatory support, affect behavioural intentions. Applying TPB in this setting, the research highlights how traditional assumptions regarding predictability in behaviour may need to be rendered sufficiently flexible to account for external uncertainties as well as informal arrangements that effectively take the place of formal institutions. The extension contributes to the literature by demonstrating that TPB remains a practical framework in risky environments, but one whose predictive power is qualified by unique contextual challenges which influence individual and organisational behaviour. This research also provides valuable insights to support East African countries in achieving the Sustainable Development Goals (SDGs), commonly called global goals.

This study provides critical theoretical implications by demonstrating that the intention of renewable energy investments is directly determined by Attitude towards renewable energy investments, subjective norm, PBC and risk aversion. The confirmation of the hypotheses (H1, H2, H3 and H4). Our results are consistent with the TPB literature in that they support the role of attitude, subjective norm, PBC, and risk aversion in defining investment intentions. In addition, the findings highlight the importance of Attitude, perceived behavioural control, subjective norms and risk aversion in shaping individuals’ Intentions to invest in renewable energy in Somalia. Furthermore, these findings expand the theoretical understanding of the interactions between Attitude, perceived behavioural control, subjective norms, risk and intention of renewable energy investments,

### Practical implications

The practical implications derived from this study suggest several strategies on promoting renewable energy investments and advance sustainable development in Somalia. The government of Somalia should implement more suitable policies related to renewable energy sources. Renewable energy investments in Somalia remain in their early stages due to a lack of knowledge and information among most investors. Given the importance of Attitude, policymakers and organizations should create awareness campaigns and educational programs targeting Attitude and perceived control towards renewable energy investments. This investigation facilitates local and international stakeholders’ understanding of the social factors influencing investment decisions. policymakers should prioritize campaigns highlighting the advantages of renewable energy, such as energy independence, cost savings, and environmental sustainability. Moreover, People in Somalia are more likely to invest in renewable energy. Thus, the Somali government should encourage private investors by establishing regulations and tax credits favourable to such investments. The governments of Somalia should give financial assistance by implementing subsidy programs and grants. The government also needs to provide financing for research and development to further the development of new technologies.

The significant influence of PBC emphasizes the importance of empowering individuals with the knowledge, skills, and resources they need to invest in renewable energy. Microfinancing, community-based renewable energy projects, and simplified investment mechanisms can improve perceived control and encourage participation. Moreover, the positive impact of perceived behaviour control on investment intention indicates that the desire to invest is increased proportionately to the degree of behavioural control shown and the ease with which it is carried out. To enhance investment intentions, it is thus more appropriate to show those with higher incomes that they can easily carry out investment activities or individuals who already have more money to invest without any constraints. This is because it is easier for these individuals to carry out investment activities. Furthermore, we suggest that individuals should have sufficient knowledge of finance or financial literacy so that individuals who understand the intricacies of the investment activities to be carried out can make effective decisions to avoid technical and financial investment mistakes. Lastly, we recommend that policymakers provide local stakeholders with practical strategies to reduce risk perceptions, such as regulatory frameworks and insurance, in light of the significant effect of risk aversion.

## Conclusion 

This research analyses investors’ intentions to invest in renewable energy in Somalia. TPB was employed as a theoretical framework for this study, focusing on intentions for renewable energy investments, which have not been thoroughly studied in prior studies. This study’s results show that the intention of renewable energy investments is directly determined by Attitude towards renewable energy investments, subjective norm, PBC and risk aversion. The findings highlight the importance of Attitude, perceived behavioural control, subjective norms, and risk aversion in shaping individuals’ Intentions to invest in renewable energy in Somalia. these results underscore the need for awareness campaigns and educational programs to foster positive perceptions of renewable energy. By addressing these factors alongside community engagement and accessible investment mechanisms, policymakers and practitioners can create effective strategies to promote renewable energy investments and advance sustainable development in Somalia.

This research focuses on individual behaviour as it relates to renewable energy investment decisions. Although this study provides good portrayals of individual intentions, attitudes, and perceived control, it does not offer an in-depth examination of the dynamics and complications at the organizational level. This represents a key limitation. Future research may also apply the analysis to organizational behaviour, as the Theory of Planned Behaviour (TPB) is applicable to examining organizational or enterprise behaviour. Future research should also consider including non-investor perspectives to obtain more generalizable results on renewable energy investment intentions in Somalia.

## Data Availability

The data supporting the findings of this study are available from the corresponding author upon reasonable request.
